# Sudan virus disease super-spreading, Uganda, 2022

**DOI:** 10.1186/s12879-024-09391-0

**Published:** 2024-05-23

**Authors:** Allan Komakech, Shannon Whitmer, Jonathan Izudi, Charles Kizito, Mackline Ninsiima, Sherry R. Ahirirwe, Zainah Kabami, Alex R. Ario, Daniel Kadobera, Benon Kwesiga, Samuel Gidudu, Richard Migisha, Issa Makumbi, Daniel Eurien, Joshua Kayiwa, Lilian Bulage, Doreen N. Gonahasa, Irene Kyamwine, Paul E. Okello, Hildah T. Nansikombi, Immaculate Atuhaire, Alice Asio, Sarah Elayeete, Edirisa J. Nsubuga, Veronica Masanja, Stella M. Migamba, Patience Mwine, Petranilla Nakamya, Rose Nampeera, Andrew Kwiringira, Rebecca Akunzirwe, Helen Nelly Naiga, Saudah K. Namubiru, Brian Agaba, Jane Frances Zalwango, Marie Gorreti Zalwango, Patrick King, Brenda Nakafeero Simbwa, Robert Zavuga, Mercy Wendy Wanyana, Thomas Kiggundu, Lawrence Oonyu, Alex Ndyabakira, Mariam Komugisha, Brian Kibwika, Innocent Ssemanda, Yasin Nuwamanya, Adams Kamukama, Dorothy Aanyu, Dominic Kizza, Daniel Okello Ayen, Sophia Mulei, Stephen Balinandi, Luke Nyakarahuka, Jimmy Baluku, Jackson Kyondo, Alex Tumusiime, Dativa Aliddeki, Ben Masiira, Esther Muwanguzi, Ivan Kimuli, Daniel Bulwadda, Herbert Isabirye, Deborah Aujo, Arthur Kasambula, Solome Okware, Emmanuel Ochien, Innocent Komakech, Charles Okot, Mary Choi, Caitlin M. Cossaboom, Carrie Eggers, John D. Klena, Modupe O. Osinubi, Katrin S. Sadigh, Mary C. Worrell, Amy L. Boore, Trevor Shoemaker, Joel M. Montgomery, Susan N. Nabadda, Michael Mwanga, Allan N. Muruta, Julie R. Harris

**Affiliations:** 1 Uganda National Institute of Public Health, Kampala, Uganda; 2 Clarke International University, Kampala, Uganda; 3 United States Centers for Disease Control and Prevention, Atlanta, GA USA; 4 Department of Community Health, Faculty of Medicine, Mbarara University of Science and Technology (MUST), Mbarara, Uganda; 5 Data Science and Evaluations Unit, African Population and Health Research Center, Nairobi, Kenya; 6 Kikandwa Health Center III, Kassanda, Uganda; 7 National Public Health Emergency Operations Center, Kampala, Uganda; 8 BAYLOR Uganda, Kampala, Uganda; 9 Kampala Capital City Authority, Kampala, Uganda; 10 Uganda Virus Research Institute, Entebbe, Uganda; 11 Department of Biosecurity, Ecosystems, and Veterinary Public Health, Makerere University, Kampala, Uganda; 12 Africa Centres for Disease Control and Prevention, Addis Ababa, Ethiopia; 13 African Field Epidemiology Network, Kampala, Uganda; 14 World Health Organization, Geneva, Switzerland; 15 Infectious Diseases Institute, Kampala, Uganda; 16 Ministry of Health, Kampala, Uganda; 17 United States Centers for Disease Control and Prevention, Kampala, Uganda; 18 National Health Laboratory and Diagnostic Services, Kampala, Uganda

**Keywords:** Ebola, Super-spreaders, Sudan virus disease, Uganda

## Abstract

**Background:**

On 20 September 2022, Uganda declared its fifth Sudan virus disease (SVD) outbreak, culminating in 142 confirmed and 22 probable cases. The reproductive rate (R) of this outbreak was 1.25. We described persons who were exposed to the virus, became infected, and they led to the infection of an unusually high number of cases during the outbreak.

**Methods:**

In this descriptive cross-sectional study, we defined a super-spreader person (SSP) as any person with real-time polymerase chain reaction (RT-PCR) confirmed SVD linked to the infection of ≥ 13 other persons (10-fold the outbreak R). We reviewed illness narratives for SSPs collected through interviews. Whole-genome sequencing was used to support epidemiologic linkages between cases.

**Results:**

Two SSPs (Patient A, a 33-year-old male, and Patient B, a 26-year-old male) were identified, and linked to the infection of one probable and 50 confirmed secondary cases. Both SSPs lived in the same parish and were likely infected by a single ill healthcare worker in early October while receiving healthcare. Both sought treatment at multiple health facilities, but neither was ever isolated at an Ebola Treatment Unit (ETU). In total, 18 secondary cases (17 confirmed, one probable), including three deaths (17%), were linked to Patient A; 33 secondary cases (all confirmed), including 14 (42%) deaths, were linked to Patient B. Secondary cases linked to Patient A included family members, neighbours, and contacts at health facilities, including healthcare workers. Those linked to Patient B included healthcare workers, friends, and family members who interacted with him throughout his illness, prayed over him while he was nearing death, or exhumed his body. Intensive community engagement and awareness-building were initiated based on narratives collected about patients A and B; 49 (96%) of the secondary cases were isolated in an ETU, a median of three days after onset. Only nine tertiary cases were linked to the 51 secondary cases. Sequencing suggested plausible direct transmission from the SSPs to 37 of 39 secondary cases with sequence data.

**Conclusion:**

Extended time in the community while ill, social interactions, cross-district travel for treatment, and religious practices contributed to SVD super-spreading. Intensive community engagement and awareness may have reduced the number of tertiary infections. Intensive follow-up of contacts of case-patients may help reduce the impact of super-spreading events.

## Introduction

Ebola disease (EBOD) is a viral haemorrhagic fever with a 25–90% case fatality rate, depending on the ebolavirus species and clinical context [1–3]. There are four viruses of the genus *Ebolavirus* known to cause EBOD infections in humans: Ebola virus (EBOV), Sudan virus (SUDV), Taï Forest virus, and Bundibugyo virus [4]. Human-to-human transmission occurs through direct contact with the blood or other body fluids of a patient with EBOD, or with the body of a person who has died of EBOD [5, 6]. Transmission can also occur after contact with infectious droplets or fluids on clothes, bedding, or medical equipment [6].

The basic reproduction number (R_o_) for EBOD, or the mean number of secondary infections produced by a typical EBOD case in a susceptible population [7, 8], is between 1.5 and 3.6 [9–13], depending on the causative virus. For SUDV (species *Sudan ebolavirus*), the basic reproduction number is estimated to be 1.3–2.7 [11, 12, 14]. During many infectious disease outbreaks, there are a small number of people who infect a disproportionate number of other persons. These persons are often referred to as ‘super-spreaders’ [15–21]. Evidence from the 2014–2016 *Zaire ebolavirus* outbreak in West Africa shows that super-spreader persons (SSPs), who comprised only 3% of cases, were responsible for up to 61% of all infections [22]. This ability to spread EBOD disproportionately to others has previously been suggested to be linked to increased strain virulence, higher pathogen shedding by hosts, differences in host-pathogen relationships, and socio-behavioural exposures [23].

On 20 September 2022, the Uganda Ministry of Health (MoH) declared the fifth Sudan virus disease (SVD) outbreak in the country [24]. The outbreak started in Mubende District and later spread to nine other districts [25]. By the end of the outbreak, 164 cases (142 confirmed and 22 probable) had been documented. The outbreak appeared to spread relatively slowly, except for two large spikes in infections in October 2022 [26]. Many patients in the large spikes were associated with two cases. The R_o_ of this outbreak was 1.25 (Kabami et al., unpublished data).

Understanding the characteristics of SSPs and identifying opportunities for transmission to large numbers of people can potentially help reduce disease spread during future outbreaks. In this paper, we present the social, clinical, and epidemiological characteristics of two SSPs from the time of their infection through outcome and describe how they contributed to the spread of SUDV during the 2022 Uganda outbreak.

## Methods

### Case investigations

The two SSPs described in this report were identified through epidemiological case investigations in Parish P, Kassanda District. We considered an SSP as a case linked to the infection of at least 13 other persons (at least 10 times the R_o_). Both had SVD confirmed using reverse-transcription polymerase chain reaction (RT-PCR). We reviewed clinical records and narratives provided by case investigation teams, who interviewed the relatives and friends of the two SSPs and other survivors who had interacted with the SSPs to understand their personal and clinical histories, disease evolution, and the degree and type of interaction with the secondary cases. Using the case-contact database, we obtained the number of contacts listed per SSP and calculated the percentage of these contacts that became cases. We used Chainchecker, an application that visualizes, curates, and helps verify transmission chain data to visualize the SVD spread from these two SSPs [27]. A probable case was defined as any person who died from suspected SVD and had an epidemiological link to a confirmed case but was not tested and did not have laboratory confirmation of the disease. A confirmed case was defined as a suspected case with a positive laboratory result for viral ribonucleic acid (RNA) detected by RT-PCR [28].

### Comparison of viral genomes

From the 142 confirmed cases, we obtained complete sequences from 104 individuals [28]. The viral genomes were aligned using MAFFT (version x), all non-A, T, C, and G characters (including inserts, and unknown or ambiguous bases) were removed from the alignment, and the raw genetic distances were calculated using R with in-house scripts [28]. Substitution rate estimates were calculated using Bayesian substitution rate estimates and 95% highest posterior rate (HPD) estimates from [28], 2.23 × 10 − 3 (1.274–3.179 × 10 − 3 subs/site/year, 95% HPD). Specifically, we calculated the number of days between Patient B’s onset date and the collection date from a specific individual. Using this timespan, we calculated the number of mutations that would be expected to arise from the Bayesian substitution rate estimates and 95% highest posterior rate (HPD) estimates, for example: Predicted nucleotide differences from Patient B= {collection date difference}/((1/(0.001274 substitutions/site/year * 18,875 bp))*365.25 days/year). The equation was repeated using Bayesian substitution rate estimates and 95% highest posterior rate (HPD) estimates to estimate the mean and range of the number of expected nucleotide differences. The number of expected differences predicted from substitution rate estimates alone was compared to the number of observed genetic distances (in base pairs).

## Ethics

This evaluation was conducted in response to an ongoing complex public health emergency. The Uganda MoH provided administrative clearance. This activity was reviewed by the United States Centers for Disease Control and Prevention (U.S CDC) human subject review board and conducted per the applicable federal law and CDC policy.§.

§See e.g., 45 C.F.R. part 46, 21 C.F.R. part 56; 42 U.S.C. § 241(d); 5 U.S.C. § 552a; 44 U.S.C. § 3501 et seq.

## Results

The two SSPs are referred to as Patient A and Patient B. Both are confirmed case-patients who were infected by the probable case (HCW1), a 26-year-old healthcare worker who worked at Clinic M, and two other clinics, all of which were located in Kassanda District. HCW1 had an illness onset on 22 September 2022 and developed progressively worsening illness including nausea, vomiting, fever, loss of appetite, and eventually hiccups. He sought healthcare at multiple clinics, including the primary healthcare clinics he worked at. Despite his illness, HCW1 continued to work at the clinics that employed him until 3 October 2022. He traveled from Kassanda District to Mityana and Wakiso Districts on 7 October 2022 and died in Wakiso District on 8 October 2022. He is linked to four subsequent confirmed SUDV infections, including the two described below, and was buried without having a sample taken.

### Case report of patient A

**Fig. 1:**
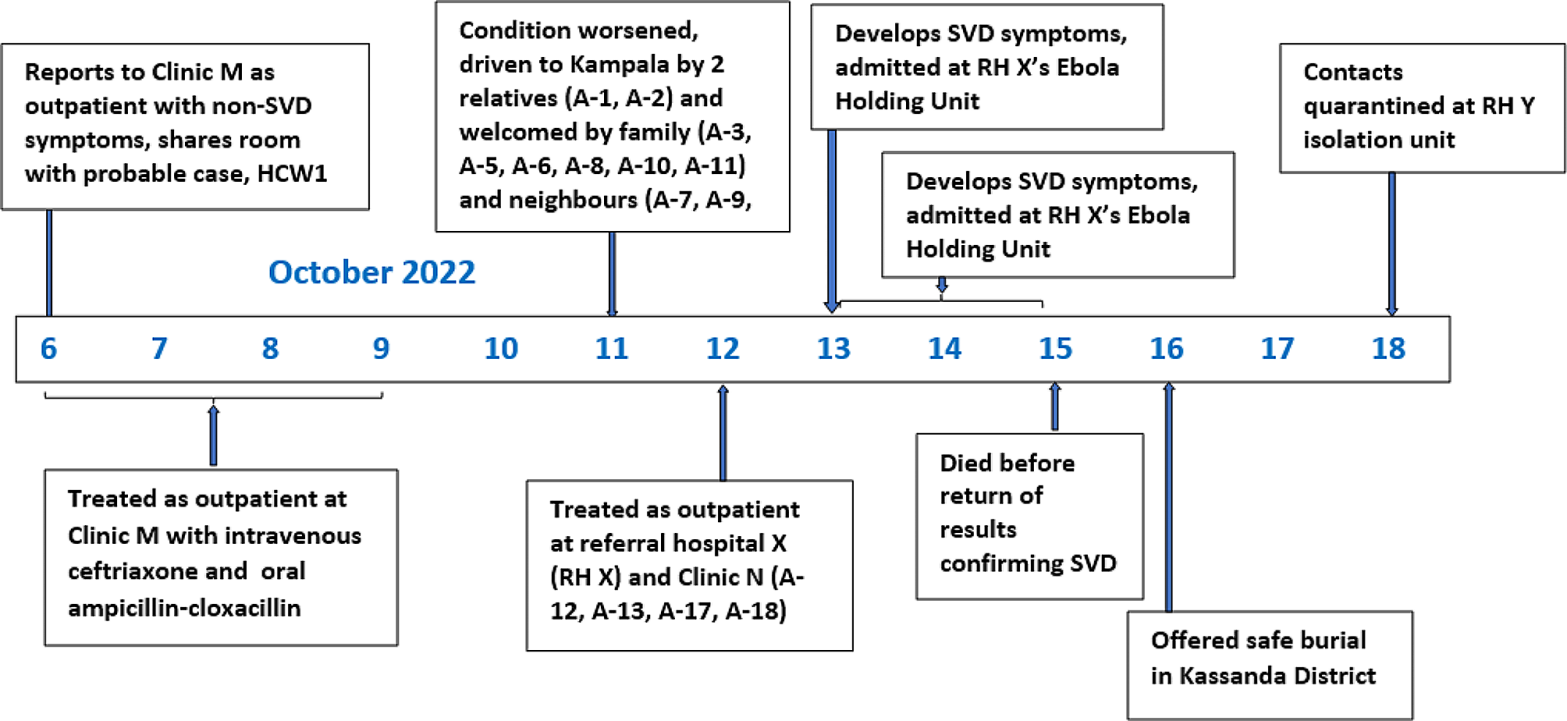
Patient A clinical presentation, health facilities visited, and secondary cases during the SVD outbreak, Uganda, 2022

Patient A was a 33-year-old male farmer and resident of Parish P in Kassanda District. On 6 October 2022, Patient A developed swelling of the face and lower limbs and visited Clinic M, a five-bed, private-for-profit Health Center II (the lowest level of a health center in Uganda) in Parish P (Fig. 1). At Clinic M, Patient A had an abdominal ultrasound that revealed cystic liver and kidney disease. During his stay at Clinic M on 6 October 2022, Patient A shared a room with HCW1, who was severely ill at the time. From 6 to 9 October 2022, Patient A was given intravenous ceftriaxone as an outpatient and was then given oral Ampiclox (ampicillin-cloxacillin) due to a lack of improvement. On 11 October 2022, his brother (case A-1, onset 17 October 2022) and another family member drove him to Kampala for more advanced clinical management. In Kampala, Patient A went to stay at another brother’s home (case A-3, with onset 16 October 2022) in Parish Q. He was accompanied in the vehicle by his brother (A-1) and stepmother (case A-2, onset 15 October 2022). The brother at whose home he stayed in Kampala (A-3), his wife (case A-10, onset 17 October 2022), and four children (A-5, A-6, A-8, A-11, all with onsets 17 October 2022) welcomed Patient A on arrival on October 11. A 4-year-old neighbor (A-7, onset 17 October 2022) and his mother (A-16, onset 18 October 2022), as well as another 12-year-old neighbor child (A-9, onset 19 October 2022) also welcomed Patient A. While at home, Patient A’s brother, A-3, and his sister-in-law, A-10, acted as his caretakers.

On 12 October 2022, Patient A presented to referral hospital X (RH X) in Kampala. He spent approximately three hours in the emergency ward and was referred for another abdominal ultrasound at a nearby private clinic (Clinic N). Case Patient A spent approximately two hours at Clinic N, where he was treated by a nurse and a sonographer, both of whom subsequently became confirmed cases (A-12 and A-13, both with onsets on 17 October 2022). The abdominal ultrasound was interpreted at RH X as being consistent with peptic ulcer disease, and Patient A was given oral omeprazole, metronidazole, and amoxicillin, and sent back to his brother’s home (A-3) in Parish Q, Kampala.

On 13 October 2022, Patient A developed bloody diarrhea and blood-stained vomitus. At this point, his family suspected that he might have SVD. They returned to RH X Emergency Ward, where staff sent him to an ‘Ebola holding unit’ inside the hospital’s emergency ward. The holding unit was a single room with five beds (three beds on one side of the room and two on the other side) used for suspected SVD cases awaiting test results or for confirmed cases awaiting evacuation to the ETU. Patient A’s blood sample was drawn on admission and sent to the MoH mobile laboratory in Mubende District for testing for SUDV. At the time of Patient A’s admission to the holding unit, there were two additional suspected SVD cases in the holding unit, each with a caretaker who slept on the ground next to their charges. Patient A stayed at the holding unit with his brother A-3, who acted as his caretaker. On 15 October 2022, Patient A died just before the release of laboratory results confirming his infection.

A 14-year-old female (case A-15, with onset 20 October 2022) was admitted on October 14 with epistaxis and placed on a bed in the holding unit approximately two meters away from the bed where Patient A was admitted. Two blood samples were taken on 14 and 17 October 2022; results were unavailable for the first sample and were negative for the second. By October 18, her epistaxis had resolved. However, on 20 October 2022, she developed diarrhea and vomiting, and a third blood sample taken from her on 21 October 2022 tested positive for SUDV.

On 16 October 2022, the body of Patient A was returned to Kassanda District and he was buried in his home village in a safe and dignified burial. On 17 October 2022, following a government policy to quarantine asymptomatic individuals in Kampala District, the caretakers and family members of Patient A in Parish Q, Kampala, all asymptomatic, were quarantined at RH X, where they stayed until 20 October 2022. On 21 October 2022, all were transferred to the Referral Hospital Z (RH Z) isolation unit.

Three additional patients were thought to have been infected by Patient A. A 22-year-old woman (A-4), who was a caretaker for one of the two patients admitted in the same ‘Ebola holding unit’ with Patient A, fell ill on 18 October 2022. She had reportedly sat on Patient A’s bed and slept on the ground one meter away from the bed of Patient A. Case A-4 tested positive on 22 October 2022. The person who she was taking care of tested negative for SUDV and was released. The second patient, a 22-year-old female from Masaka District (case A-17) was admitted on 31 October 2022 to Masaka Regional Referral Hospital with a haemorrhage from the nose and vomiting blood. She had onset on 21 October 2022 and experienced a spontaneous abortion on 28 October 2022. On November 1, 2022, a blood sample from A-17 tested positive for SUDV. She died on 2 November 2022 while being evacuated to the ETU. Unfortunately, she died before she could be interviewed. A review of clinical records and interviews with her husband revealed that she had been both at Clinic N and at RH X on 12 October 2022, the same day that Patient A visited those sites. Genetic sequencing showed that the virus isolated from Patient A was closely related to A-17, with 2 nucleotides difference between them (the expected mutation rate for viruses isolated from sequential patients approximately 3 weeks apart) (Table 1).

A third patient linked to Patient A, probable case A-18, was a 39-year-old male traditional healer living and working only a few hundred meters from RH X. He had onset of headache and flu-like symptoms on 20 October 2022, and sought care at both Clinic N and a RH X clinic on 23 October 2022. His previous exposures at either the clinic or to Patient A are unknown. He traveled from Kampala to Jinja on 27 October 2022 and died on 28 October 2022 in Jinja. He was linked to two confirmed SVD cases. While no sample was taken from A-18, he subsequently infected his brother, A-18-1, for whom a viral sequence is available. SUDV isolated from A-18-1 had only a single nucleotide difference from Patient A, suggesting that they are closely linked and that Patient A was the likeliest source of infection for A-18 (Table 1).

Genetic sequencing of Patient A and his close contacts supported most of the epidemiological connections. A comparison of 16,528 shared nucleotides (out of 18,875 total base pairs) demonstrated that Patient A had genomes within or close to the predicted substitution rate range with A-1, A-2, A-10, A-12, A-13, A-14, A-15, A-17, and A-18-1 (Table 1). Cases A-4 and A-16 exhibited more mutations than would be predicted by the substitution rate, making their direct linkage to Patient A less certain; their linkages are based on epidemiologic connections (Table 1). Genomic sequences were not available for A-5, A-8, A-11, or A-18 due to low concentrations of the virus in blood samples.

Overall, Patient A is suspected to have infected 18 people (16 in Kampala City and 2 in Kassanda District) (Fig. 1); eight were family members, three were neighbors, and seven were persons with whom he came into contact at different health facilities. Among the 18 secondary cases, four (22%) were believed to have had indirect exposure to Patient A, and the others (14; 78%) had direct exposure. Of the 18 secondary cases, four died (A-17 in Masaka District, A-18 in Jinja District, and A-9 and A-16 in Kampala). Two tertiary cases, A-18-1 and A-18-2, both relatives of A-18, were reported from the networks of the 18 people with secondary infections. The travel of Patient A outside of Kassanda District and the resultant exposures in Kampala led to the implementation of a lockdown in Kassanda and Mubende districts starting on 15 October 2022, when Patient A’s infection was confirmed, to prevent further spread from the affected districts.

**Table 1:** Genetic distances between Patient A and secondary cases linked to exposure to Patient A during the Sudan virus disease outbreak in Uganda, 2022. Only secondary with sequence data linked to Patient A (*n* = 11) and Patient A-18 (*n* = 1) are shown

	Collection Date difference from Patient A	Predicted nucleotide differences from Patient A	Patient A	A-1	-2	A-4	-9	A-10	-12	A-13	-14	A-15		-16	A-17
Patient A															
A-1	6	0.7 [0.4-1.0]	0												
A-2	6	0.7 [0.4-1.0]	0	0											
A-4	7	0.8 [0.5–1.1]	4	4	4										
A-9	8	0.9 [0.5–1.3]	0	0	0	4									
A-10	8	0.9 [0.5–1.3]	0	0	0	4	0								
A-12	8	0.9 [0.5–1.3]	2	2	2	6	2	2							
A-13	8	0.9 [0.5–1.3]	0	0	0	4	0	0	2						
A-14	19	2.2 [1.3–3.1]	0	0	0	4	0	0	2	0					
A-15	10	1.2 [0.7–1.6]	1	1	1	5	1	1	3	1	1				
A-16	10	1.2 [0.7–1.6]	4	4	4	8	4	4	6	4	4	3			
A-17	17	2.0 [1.1–2.8]	3	3	3	7	3	3	5	3	3	4	7		
A-18-1	28	3.2 [1.8–4.6]	1	1	1	5	1	1	3	1	1	2	5		4

### Case report of patient B

**Fig. 2:**
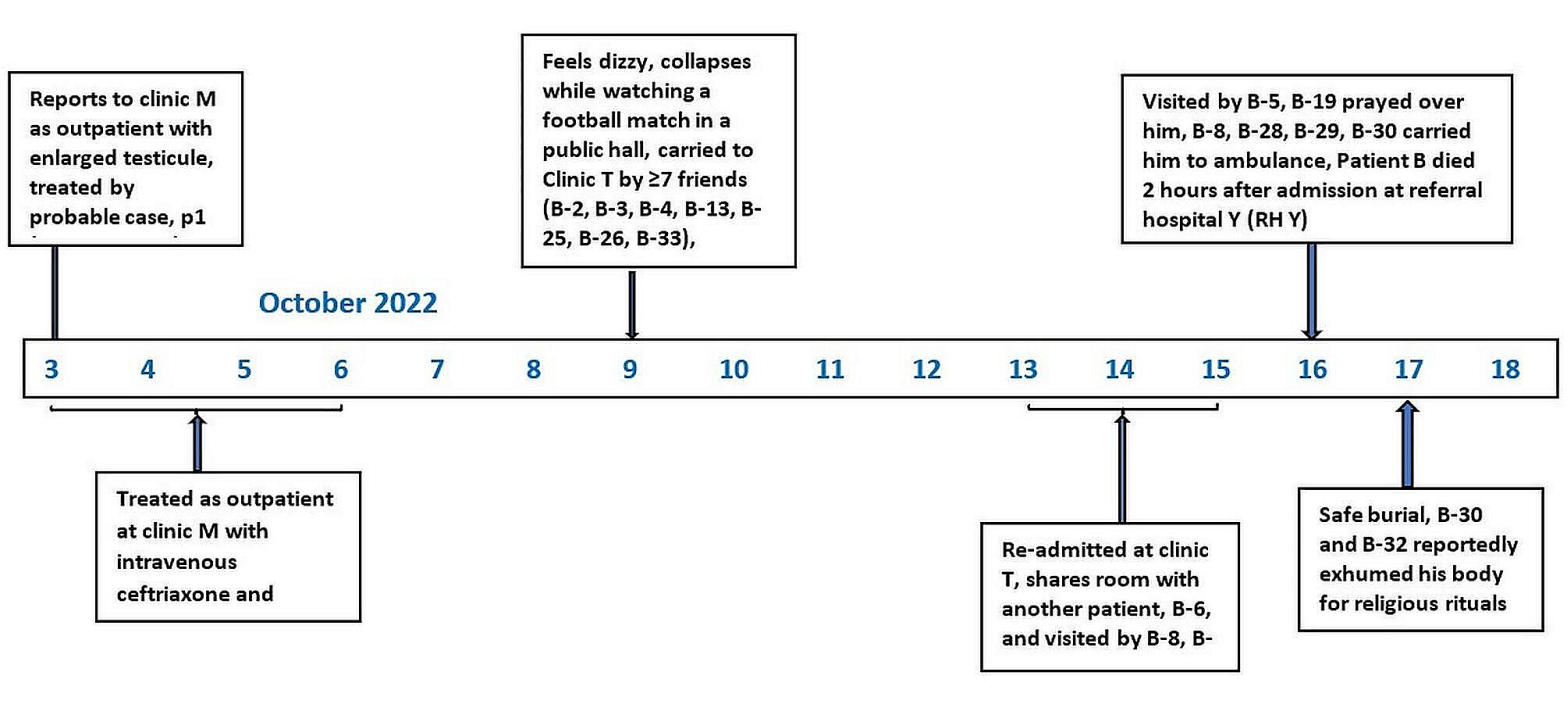
Patient B’s clinical presentation, health facilities visited, and secondary cases during the SVD outbreak, Uganda, 2022

Patient B was a 26-year-old male butcher and a resident of Parish P in Kassanda District. He reportedly had an active social life with many of the young people in the community. Patient B first presented at Clinic M on 3 October 2022, with an enlarged testicle (Fig. 2). He was seen by HCW1, who was ill but working. He diagnosed Patient B with acute orchitis and gave him intravenous ceftriaxone and metronidazole from 3 to 5 October 2022, which Patient B received as an outpatient.

On 9 October 2022, while watching a football match with friends in a public hall in Parish P, Patient B developed dizziness and headache. He left the hall and collapsed in the street. At this point, he was taken to the nearby private Clinic T by several friends who were watching the match with him. Clinic T is a private-for-profit Health Centre II in Parish P that offers both outpatient and inpatient services. Patient B was reportedly assisted in reaching the clinic by at least 7 friends (B-2, B-3, B-4, B-13, B-25, B-26, B-33; onsets October 16–22) who subsequently tested positive for SUDV. Case Patient B was received by a healthcare provider, and later confirmed as an SVD case (B-5, onset 18 October 2022). At Clinic T, Patient B reported extreme weakness. He tested negative for malaria and was diagnosed with typhoid fever. He was prescribed oral amoxicillin and was taken back home by his friends.

From 13 to 15 October 2022, Patient B was re-admitted at Clinic T. During this time, he shared a room with a 6-year-old girl (case B-6; onset 21 October 2022) who sought treatment at Clinic N for a gastrointestinal illness during 15–16 October 2022; she tested positive for SUDV on 23 October 2022. During Patient B’s stay at Clinic T, he was also visited by several friends, of whom five tested positive (B-8, B-12, B-14, B-16, B-21; onsets 18–23 October). On 15 October 2022, due to a lack of improvement, Patient B and his family were advised by the healthcare provider (B-5) to seek higher-level care at referral hospital Y (RH Y). He was taken back home late that evening and spent the night at home, where he was visited by several relatives and friends.

On 16 October 2022, Patient B was visited at home by the healthcare provider B-5, who found relatives and friends in close contact with Patient B. Suspecting that Patient B had SVD, another visiting healthcare worker called the SVD alert line to request an ambulance. However, the ambulance was delayed, and Patient B spent almost the entire day at home, visiting relatives, friends, and religious leaders gathering and praying over him. Among those who had direct contact while praying with him, B-19 (onset 20 October 2022) subsequently tested positive for SUDV. That evening, Patient B began bleeding from the nose. Due to the delay in the arrival of the ambulance, the family attempted to send him to the hospital on a motorcycle taxi, commonly known as *“boda-boda”*, late in the evening. However, while being assisted onto the boda-boda, Patient B reportedly convulsed and collapsed in the street. At approximately 9 pm, the ambulance arrived and transported him to RH Y’s Ebola treatment unit (ETU), where he died two hours later. It rained heavily the night of his evacuation while the patient’s family and friends helped him into the ambulance while he was covered with blood. Several of the people who assisted him (B-8, B-29, B-30; onsets 21–22 October 2022) subsequently tested positive for SVD. An ambulance nurse who participated in the evacuation of Patient B to the ETU on that day also tested positive for SVD 8 days later (B-28, onset 23 October 2022).

Patient B also infected several members of his family including his brothers (B-9 and B-15; onsets 21 and 23 October 2022), mother (B-11; onset 21 October 2022), wife (B-17; onset 23 October 2022), a neighbor who washed Patient B’s clothes while his wife was away (B-18; onset 23 October 2022), and the boda-boda driver who tried to transport him to the hospital on October 16 (B-22; onset 20 October 2022). Most of his relatives and friends had several points of contact with him during his illness in the community, and for many, the precise date of their relevant exposure was difficult to identify. When interviewed after Patient B’s death, several of his friends reported that, although they were aware of the SVD outbreak, they suspected that Patient B was bewitched by someone from whom he had stolen a goat, and thus were not concerned about contracting illness from him.

Overall, Patient B is linked to at least 33 secondary cases, of whom 14 (41%) died. Of the 33 secondary cases, 1 (3%) case was likely exposed only through washing Patient B’s clothing. Seven tertiary SVD cases were linked to persons infected by Patient B. Case Patient B was buried in a safe and dignified manner at his home in Kassanda District. There were unconfirmed rumors, denied by the family, of the exhumation of his body for a traditional Islamic burial. Among those who were reported to have exhumed his body, two (B-30 and B-32; onsets 21 and October 22 October 2022, respectively) subsequently tested positive for SUDV. Their contact with him before his death is unknown.

A blood specimen was not available for testing for Patient B, precluding genetic sequencing. However, genomes from Patient A and another patient (Patient C) infected by HCW1, which were indistinguishable from each other, were used as proxy genomes for Patient B. A comparison of 18,599 shared nucleotides (out of 18,875 total base pairs) demonstrated that viruses from Patient C and Patient A had genomes within or close to the predicted substitution rate range (0–3 nucleotides) for all of the secondary cases attributed to exposure to Patient B (Table 2).

### Analysis of time to isolation at the Ebola treatment unit of secondary cases linked to the two superspreader persons

Both Patient A and Patient B were highly suspected to have SUDV infection when they died, and the case investigation teams collected full narratives the day after their deaths. The details of these narratives provoked intensive follow-up from the response teams in Kassanda and Kampala District, including widespread community awareness creation, active case-finding, and follow-up and monitoring of contacts. Due to concerns about uncontrolled urban spread, in Kampala, contacts were pre-emptively isolated before symptom onset.

Of the 18 secondary infections from SSP Patient A, 16 (89%) were admitted to an ETU during their illnesses. All 33 persons infected by Patient B were admitted to an ETU. Among the 49 secondary cases from Patient A and Patient B who were admitted to an ETU, the median time from onset to admission was 3 days (IQR:1–7 days). The overall median time to isolation for all cases during the outbreak was 5 days (IQR:3–8 days).

**Table 2:** Genetic distances between Patient A, Patient C, and secondary cases associated with exposure to Patient B during the Sudan virus disease outbreak in Uganda, 2022. Only secondary cases with sequence data (*n* = 28) are shown

	Specimen collection date difference (days) from Patient B’s onset	Predicted nucleotide differences from Patient B	Patient A	Patient C	B-2	B-3	B-4	B-13	B-26	B-33	B-5	B-6	B-8	B-12	B-14	B-16	B-21	B-19	B-24	B-30	B-28	B-9	B-15	B-11	B-17	B-18	B-22	B-32	B-20	B-23	B-27
Patient A	9	1.03 [0.6–1.5]																													
Patient C	8	0.92 [0.5–1.3]	0																												
B-2	18	2.07 [1.2-3.0]	1	1																											
B-3	18	2.07 [1.2-3.0]	0	0	1																										
B-4	18	2.07 [1.2-3.0]	0	0	1	0																									
B-13	20	2.30 [1.3–3.3]	0	0	1	0	0																								
B-26	22	2.54 [1.4–3.6]	0	0	1	0	0	0																							
B-33	23	2.65 [1.5–3.8]	1	1	2	1	1	1	1																						
B-5	18	2.07 [1.2-3.0]	0	0	1	0	0	0	0	1																					
B-6	19	2.19 [1.3–3.1]	0	0	1	0	0	0	0	1	0																				
B-8	19	2.19 [1.3–3.1]	0	0	1	0	0	0	0	1	0	0																			
B-12	20	2.30 [1.3–3.3]	0	0	1	0	0	0	0	1	0	0	0																		
B-14	20	2.30 [1.3–3.3]	1	1	2	1	1	1	1	2	1	1	1	1																	
B-16	20	2.30 [1.3–3.3]	0	0	1	0	0	0	0	1	0	0	0	0	1																
B-21	21	2.42 [1.4–3.4]	0	0	1	0	0	0	0	1	0	0	0	0	1	0															
B-19	21	2.42 [1.4–3.4]	0	0	1	0	0	0	0	1	0	0	0	0	1	0	0														
B-24	22	2.54 [1.4–3.6]	0	0	1	0	0	0	0	1	0	0	0	0	1	0	0	0													
B-30	23	2.65 [1.5–3.8]	0	0	1	0	0	0	0	1	0	0	0	0	1	0	0	0	0												
B-28	23	2.65 [1.5–3.8]	0	0	1	0	0	0	0	1	0	0	0	0	1	0	0	0	0	0											
B-9	20	2.30 [1.3–3.3]	0	0	1	0	0	0	0	1	0	0	0	0	1	0	0	0	0	0	0										
B-15	20	2.30 [1.3–3.3]	2	2	3	2	2	2	2	3	2	2	2	2	3	2	2	2	2	2	2	2									
B-11	20	2.30 [1.3–3.3]	0	0	1	0	0	0	0	1	0	0	0	0	1	0	0	0	0	0	0	0	2								
B-17	21	2.42 [1.4–3.4]	0	0	1	0	0	0	0	1	0	0	0	0	1	0	0	0	0	0	0	0	2	0							
B-18	20	2.30 [1.3–3.3]	0	0	1	0	0	0	0	1	0	0	0	0	1	0	0	0	0	0	0	0	2	0	0						
B-22	22	2.54 [1.4–3.6]	3	3	4	3	3	3	3	4	3	3	3	3	4	3	3	3	3	3	3	3	5	3	3	3					
B-32	26	3.00 [1.7–4.3]	0	0	1	0	0	0	0	1	0	0	0	0	1	0	0	0	0	0	0	0	2	0	0	0	3				
B-20	21	2.42 [1.4–3.4]	0	0	1	0	0	0	0	1	0	0	0	0	1	0	0	0	0	0	0	0	2	0	0	0	3	0			
B-23	22	2.54 [1.4–3.6]	2	2	3	2	2	2	2	3	2	2	2	2	3	2	2	2	2	2	2	2	4	2	2	2	5	2	2		
B-27	22	2.54 [1.4–3.6]	0	0	1	0	0	0	0	1	0	0	0	0	1	0	0	0	0	0	0	0	2	0	0	0	3	0	0	2	
B-31	24	2.77 [1.6–3.9]	1	1	2	1	1	1	1	0	1	1	1	1	2	1	1	1	1	1	1	1	3	1	1	1	4	1	1	3	1

## Discussion

Exposure to two persons, both infected by a single probable SVD case, led to at least 51 secondary infections during the 2022 SVD outbreak in Uganda. These secondary infections accounted for 31% of the cases in the entire outbreak. The infections occurred across a wide range of settings and resulted from direct and indirect exposures at home, in healthcare facilities, and during burials. Despite the high numbers of secondary infections resulting from exposures to these super-spreader persons, few tertiary infections occurred.

A small number of reports describe EBOD super-spreading [23, 29–36], although none have described super-spreading due to SUDV. Outside of SSPs, the expected number of secondary cases in a susceptible population for SUDV is 1.3–2.7 cases per case [11, 12, 14], and 1.4–4.7 cases per case for EBOV [11–14, 37, 38]. Previous reports of SSPs from the West Africa outbreak, caused by EBOV, included 24 secondary cases from a single source in Sierra Leone [33], 15 secondary cases from one case in Guinea [35], and 13 cases linked to funeral practices during the burial of a traditional healer in Sierra Leone [36]. The combination of factors that produce an SSP or super-spreading event is unknown. Super-spreading has been suggested to be attributable to increased strain virulence (and presumably a lower infectious dose), higher pathogen shedding, and increased numbers of social exposures for some patients compared to others [23].

In this outbreak, the high number of contacts of both SSPs and the time they spent in the community while ill – essentially their entire illnesses - likely facilitated the high numbers of secondary infections. In Uganda, friends and relatives normally visit sick people at home, especially when they are seriously ill, and this provides ample opportunity to transmit a virus such as SUDV, especially late in illness when a patient is experiencing ‘wet’ symptoms. Praying over ill persons in Uganda typically involves touching the sick person [39], which can serve as a source of exposure. Wong et al. (2015) [30] and Mohindra et al. (2021) [31] reported that behavioural interactions in the community, such as caring for the sick and regular visits by relatives that result in overcrowding at home are frequently associated with super-spreader events. However, this is unlikely to fully explain the SSP. During the outbreak, three other confirmed cases spent their entire illnesses in the community (Kabami et al., manuscript submitted) yet were not associated with high numbers of secondary infections. Additional factors beyond the time spent in the community contribute to the likelihood of super-spreading.

Data generally suggest that direct contact (direct contact with blood or body fluids of an infected person with symptoms or a dead body), as opposed to indirect contact such as exposure to fomites, is the most important mechanism of transmission in an EBOD outbreak [40–42]. In this outbreak, 5 of the 51 secondary cases from the two SSPs were likely infected through fomite exposure. The healthcare-associated infection of A-15 was likely caused by contact with the bedding of Patient A in the holding unit. Case B-18’s only reported exposure to Patient B was washing his clothing. The exposures of cases A-17 and A-18 may have been contact with surfaces in the same clinic or physical area as Patient A; however, this is speculative as both patients died without being interviewed. Case A-4 was also thought to be infected through exposure to the bedsheets of Patient A; however, both A-4 and another patient (A-16) had sequence data that made a direct link to Patient A less certain than the links to the other patients linked him, despite epidemiologic links and no other known exposures. Specifically, A-16 is part of a family and group of neighbours whose infections were all directly linked, both epidemiologically and genetically, to Patient A, while A-4 took care of a patient who tested negative in the SVD holding ward but was isolated together with Patient A. It is possible that there were errors in their sample-taking, sample labeling, and sample sequencing, or that there are other, unidentified sources of their infections. Because of this, Patient A remains the most likely source of infection for these patients.

Socio-cultural and religious beliefs in the community, including those surrounding witchcraft and ancestral spirits, may have contributed to the delays in the identification of infection and the subsequent isolation of Patient B. Their symptoms were initially linked to witchcraft and ancestral spirits, factors that can propagate transmission as reported during previous EBOV outbreaks [43–49] and this outbreak (Nelly et al., manuscript in preparation). Sociocultural beliefs may lead to a failure to follow preventive measures, or the seeking of alternative, non-traditional care for EBOD. Both can cause delays in diagnosis and isolation, creating opportunities for further spread [39]. Despite the outbreak being established at the time of Patient B’s illness and community awareness about the outbreak, the belief that Patient B was bewitched for having stolen a goat reduced community suspicion about his having SVD. Failure of safe and dignified burials to adhere to specific cultural burial rituals has also previously been implicated in exhumations of EBOD patients, where it was also reported as a source of spread [50, 51].

A previous study on spatial and temporal dynamics during the 2014–2015 EBOV outbreak in West Africa suggests that an individual’s social contact structure contributes greatly to the spread of EBOD [29]. The study particularly suggests that age-dependent social structure might be an important factor in superspreading, with possible theories related to the young and old having more visits and caretakers compared to other age groups [29]. Narratives from the community indicated that Patient B was highly social and active in the community. He also continued to interact even while feeling unwell and had large numbers of people around him. His sudden collapse attracted help from the community, which likely led to the infection of several other people. Furthermore, visits at clinics from friends and family and at home while Patient B was ill especially immediately before he died almost certainly contributed to his disease spread. This emphasizes the need to study social structures where specific groups may be identified as having a wider social structure than others, and therefore prompting targeted interventions [29]. However, in cases where it is impossible to study social structures, aside from the known interventions such as educational outreaches and community awareness, it is also crucial to limit visitation rights at all hospitals/clinics or other households within an outbreak area, measures that have previously not been discussed in the literature.

EBOD super-spreaders, by definition, cannot be identified until the infections have already manifested among their contacts [30]. While the large-scale spread of infectious diseases can be reduced to some extent through the prohibition of community events that might facilitate such spread, it is difficult to prevent the first generation of super-spreading. However, attention to specific response activities can reduce the risk of tertiary infections from patients with high numbers of exposed contacts or high-risk exposure events, such as occurred during the last day of Patient B’s life. The intensive community engagement and the strong follow-up of contacts of Patients A and B were likely critical factors in reducing the spread from the secondary cases to tertiary cases. Other interventions, such as geographic lockdowns, could also be warranted when high numbers of infections are expected through such exposures. The impact of combined measures to curb EBOD spread has been well-documented in previous outbreaks [52].

Our study has three main limitations. First, there is some lack of clarity in the timeline of exposure for Patient A. A time from SUDV exposure to death of nine days is unusual but not impossible; typically, at least two weeks elapse from exposure to death for SDV patients [40, 53]. The narratives collected about Patient A identified no other point before 6 October 2022 during which he would have been exposed to a SUDV patient; however, since information was not available about him until after his death, it is possible that some details were not known by those who reported or were not available in the medical records. The onset date of the health problems for which Patient A initially sought care at Clinic M is unclear, and it is possible that the problems that brought him to Clinic M were related to a SUDV infection acquired earlier and from another source, or perhaps from an earlier, unreported exposure to CHW1. It is also possible that the existing illness in Patient A that brought him to Clinic M also accelerated the progression of his SUDV illness and resulting death. The two elements that strongly suggest that CHW1 was the source of Patient A are the room-sharing on 6 October 2022, and the genomic sequencing data that link Patient A and nearly all of the secondary cases resulting from exposure to Patient B, also infected by CHW1. As such, the infection in Patient A is strongly suspected to result from exposure to CHW1. A second, equally important limitation of our study was a lack of timely adoption of genetic sequencing on viruses from SVD cases. Although genetic sequencing provided evidence to support the contact tracing chains, the sequence data were not available for some cases, and retrospective analysis was only conducted timely once the adoption of genome sequencing had been fully embraced. While no closer genetic links to A-4 and A-16 to Patient A were identified among the patients whose viruses were sequenced, these two patients may have had exposures that were not captured and be linked to other patients whose viruses were not sequenced. Third, although our study identified several social and epidemiological characteristics of our SSPs that might have led to superspreading, we were unable to exhaustively identify other characteristics known to be associated with superspreading such as increased strain virulence and higher pathogen shedding. Our results should therefore be interpreted in this context.

## Conclusion and recommendations

Two super-spreader persons were identified during the SVD outbreak in Uganda, contributing to nearly 1 in 3 infections during the outbreak. The super-spreading was facilitated by high levels of interaction of patients during their long stay in the community while ill, patient travel, traditional sociocultural beliefs and religious practices as well a large social network of friends and family. Sequencing data were critical for confirming suspected epidemiologic links between cases. Few tertiary cases were reported from the networks of Patient A and Patient B respectively. We recommend a strong focus on the early identification of all suspected EBOD cases who present with both typical and atypical symptoms and their immediate isolation at the local level to prevent population exposure and reduce the risk of such events. We also recommend the adoption of timely and robust sequencing data early during outbreaks to inform disease transmission dynamics. Future studies should also be done to evaluate the role of social network size on super-spreading events.

## Data Availability

The personal details datasets used for this descriptive analysis are a property of the WHO and Uganda MoH and are not publicly available. However, with a reasonable request and permission from WHO and Uganda MoH, data can be accessed through the corresponding authors. The genomes used in the current study are available on Genbank, with accession numbers OQ672950–OQ673069 [28].

## Abbreviations

EBOD: Ebola disease
EBOV: Ebola virus (species *Zaire ebolavirus*)
MoH: Ministry of Health
R_o_: Basic reproduction number
RNA: Ribonucleic acid
RT-PCR: Reverse transcription polymerase chain reaction
SSP: Super-spreader person
SUDV: Sudan virus
SVD: Sudan virus disease
U.S CDC: United States Centers for Disease Control and Prevention
